# Cell-free DNA From Pleural Effusion Samples: Is It Right for Molecular Testing in Lung Adenocarcinoma?

**DOI:** 10.3389/pore.2021.613071

**Published:** 2021-03-30

**Authors:** Attila Mokánszki, Emese Sarolta Bádon, Anikó Mónus, László Tóth, Nóra Bittner, Gábor Méhes

**Affiliations:** ^1^ Department of Pathology, Faculty of Medicine, University of Debrecen, Debrecen, Hungary; ^2^ Department of Pulmonology, Faculty of Medicine, University of Debrecen, Debrecen, Hungary

**Keywords:** lung carcinoma, pleural effusion fluid, liquid biopsy, cytological cell block, mutation analysis

## Abstract

Pathogenic molecular features gained specific significance in therapeutic decisions in lung carcinoma in the past decade. Initial and follow up genetic testing requres appropriate amounts and quality of tumor derived DNA, but tumor sampling, especially for disease monitoring is generally limited. Further to the peripheral blood (PB), samples from pleural fluid, accumulating in diverse lung processes might serve as an alternative source for cell-free DNA (cfDNA) for genetic profiling. In our study, cfDNA isolated from the pleural effusion and from the PB, and genomic DNA (gDNA) obtained from tissue/cellular samples were analyzed and compared from altogether 65 patients with pulmonary disease, including 36 lung adenocarcinomas. The quantity of effusion cfDNA yield appeared to be significantly higher compared to that from simultaneously collected PB plasma (23.2 vs. 4.8 ng/μl, *p* < 0.05). Gene mutations could be safely demonstrated from the effusion cfDNA fraction obtained from adenocarcinoma patients, 3/36 *EGFR*, 9/36 *KRAS* and 1/36 *BRAF* gene variants were detected. In this series, 9/13 samples showed an effusion+/plasma-mutational status, while only 1/13 samples presented with the opposite findings (effusion-/plasma+). gDNA analysis from sediment cell blocks from the identical effusion sample was surprisingly ineffective for lung adenocarcinoma profiling due to the low DNA yield. In conclusion, the cell free supernatant of pleural effusions appears to concentrate cancer derived cfDNA and seems to be particularly suitable for serial genotyping of pulmonary adenocarcinoma.

## Introduction

The expanding number of targeted treatment modalities inferred extensive predictive molecular testing in lung cancer [1]. DNA based analysis of activating mutations in the *EGFR*, *KRAS* and *BRAF* genes became a prerequisite for the efficacy of anti-EGFR therapy. Unfortunately, lung tumor tissue biopsy is sometimes technically challenging or samples are inadequate due to sampling error or low tumor cell content [2]. Moreover, repeated sampling is increasingly required to monitor therapeutic efficacy or resistance related changes, which is hardly tolerable for the patient. Therefore, there is a growing need for non-invasive alternative sources of tumor-derived DNA. The circulating cell-free DNA (cfDNA) fraction of the peripheral blood (PB) plasma appears to represent tumor derived DNA fragments and enables the genetic analysis from a single tube of *p*B. In agreement with the high expectations to replace tissue sampling this approach is also frequently called liquid biopsy [3–7]. Unfortunately, many variables influence the actual amount and composition of the extractable cfDNA, and blood liquid biopsies may remain unsuccesful in up to 50% of the samples [8].

Pleural effusions develop as transudates in many disorders involving the pleura or the lung parenchyma [9, 10]. Probably the most important clue is the exclusion of its neoplastic origin, especially if the pleural cavity is unilaterally involved. Above 75% of the malignant pleural effusions are observed in association with advanced malignancies of the lung, including primary pulmonary cancer and metastatic tumors. Patients with pleural effusate are regularly treated with thoracic puncture resulting fluid samples. Cytology examination of the punctured fluid is subject of cytomorphological examination to demonstrate the extent and quality of the neoplastic involvement in the pleural space. However, the sensitivity of cytological examinations is contradictory [11, 12].

The preparation of the pleural effusion by centrifugation results two fractions: sediment cells – used primarily for cytology - and a cell-free fluid component traditionally considered as waste. However, with the development of the technology further testing of minute amounts of cell-free nucleic acid fraction is made possible. The effusion fluid is anatomically close to the diseased lung parenchyma and its production is strongly related to the actual lymphatic circulation. In theory, the aberrant lymphatic drainage of the tumor area directly contributes to pleural fluid, consequently supplying with high tumor derived cfDNA quantities [13]. Clinically significant pleural effusions may be present and can be collected from the earliest stage of lung malignancy. Therefore, occurrance of tumor derived cfDNA in the pleural space may preceed morphologically significant tumor cell quantities and may also present with higher quality for molecular testing compared with blood plasma. On the contrary, similar to the cellular fraction the precipitate might be highly heterogeneous, including nucleic acid fragments from non-neoplastic mesothelial or inflammatory cells, influencing both sensitivity and specificity.

The goal of our prospective examination was 1) to test the utility of cfDNA isolated from pleural effusion samples in patients with lung disease, including adenocarcinoma, 2) to analyze correlations between pleural effusion and plasma cfDNA amounts and quality, 3) to prove the relationship between pleural fluid cfDNA and sediment cells DNA, 4) to identify and compare relevant pathogenic gene variants (*EGFR*, *KRAS* and *BRAF*) in cfDNA originated from pleural effusion and blood and 5) compare the cfDNA based molecular profile with the tumor tissue and the effusion sediment cell derived gDNA data. For this purpose cfDNA and gDNA were isolated and analyzed from all available samples of the same patients (lung tissue biopsy, effusion sediment cells, cytology smear, blood plasma and acellular pleural effusion fluid) using a simple high sensitivity strip-based reverse hybridization assay.

## Materials and Methods

### Study Cases and Samples

Patient population derived from the Department of Pulmonology, University of Debrecen from the period of November 2019–September 2020. Samples from 65 patients with hydrothorax of variable origin undergoing therapeutic drainage of pleural effusion were included in the study. cfDNA (effusate supernatant) and gDNA (effusate sediment) from the same sample were isolated from all 65 effusate sample. In 29 cases matched lung tissue biopsy and in thirteen cases blood plasma samples were available at the same time for comparative analysis. In five cases cytological smears complemented the study, as sediment cell block could not be prepared due to the limited amount of cells in pleural effusion.

50 ± 0.5 ml pleural effusion fluid was centrifuged at 1,500 g for 5min resulting sediment cells (used for FFPE cell blocks) and cell-free supernatant. Supernatant fracions were repeatedly spinned down (16,000 g, 10 min) to eliminate cell residues. 5 ± 0.5 ml blood plasma served as a paired control for the analysis. Blood samples were taken in EDTA anticoagulant tubes and were centrifuged at 3,000 g for 10 min repeated by another centrifugation as described earlier. Formaldehyde fixed and paraffin embedded (FFPE) lung tissue biopsy samples were processed according to the institutional routine diagnostic procedures.

All study subjects have been endorsed by Institutional Review Board (4941/2018). Patients were informed and consent was given. This study was managed according to the Declaration of Helsinki.

### DNA Isolation

CfDNA isolation was carried out from 5 ml supernatant of the pleural effusion fluid and/or 5 ml blood plasma samples using QIAamp Circulating Nucleic Acid Kit (Qiagen, Hilden, Germany). DNA isolation from sediment cell blocks and cytological samples was performed using QIAamp DNA Mini Kit (Qiagen, Hilden, Germany). Genomic DNA from FFPE tumor biopsy samples was isolated using QIAamp DNA FFPE Tissue Kit (Qiagen, Hilden, Germany). 50 µl elution buffer served for the DNA dilution. Qubit dsDNA HS Assay Kit was used to determine DNA concentration using a Qubit 4.0 Fluorometer (Thermo Fisher Scientific, Waltham, MA, United States). The distribution and fragment size estimation of double stranded cfDNA deriving from effusates was done using a Bioanalyzer 2100 device (Agilent Technologies, Santa Clara, CA, United States).

### Mutation Detection by Reverse-Hybridization Assay (StripAssay)

For sensitive mutation detection the reverse-hybridization technology was chosen using *EGFR* XL, *KRAS* XL and *BRAF* 600/601 reverse hybridization strips (StripAssay, ViennaLab Diagnostics, Vienna, Austria). The reaction volumes were 20 µl, with an optimal input of 50 ng DNA per sample independent of their origin. The reaction protocol was performed according to the manufacturer. 30 clinically relevant pathogenic variants are covered by the *EGFR* XL, 29 mutations by the *KRAS* XL and 9 by the *BRAF* XL strips. The protocol is certified for human *in vitro* diagnostics (IVD). This assay is capable of detecting variants >1% in the DNA samples. The results were determined and interpreted by the evaluation template supplied with the kits.

### Statistical Analysis

SPSS software was used for the statistical analysis (SPSS Inc., Chicago, IL, United States). Variables were compared with the Student’s t-test and Pearson correlation analysis was used to acess correlations. *p* < 0.05 was considered significant.

## Results

### Patients and Samples

Altogether 65 patients samples were studied, 38 were male (58.46 %) and 27 (41.54 %) were female. The average age was 67 years, ranging from 25 to 87. The clinicopathological characteristics of the study population is summarized in Table 1.

**TABLE 1:** Clinical characteristics of patients (MPE: malignant pleural effusion, BPE: benign pleural effusion).

	MPE (*n* = 41)	BPE (*n* = 24)	Total (*n* = 65)
**Age (years)**			
Average	65.2	70.6	67
Range	25–83	36–87	25–87
**Sex**			
Male	22 (53.7%)	16 (66.7%)	38
Female	19 (46.3%)	8 (33.3%)	27
**Pleural fluid cytology**			
Positive for malignancy	22 (53.7%)	0%	22 (33.8%)
Negative for malignancy	19 (46.3%)	100%	43 (66.2)

The terminology of malignant pleural effusion (MPE) was used if the pleural sample was associated with a malignant finding (fluid proved to be positive for malignancy or, negative fluid matched an independent malignant lung tissue biopsy/cytology). Benign pleural effusions (BPE) were stated if pleural fluid and lung cytology, histology and the clinical appearance all exluded malignant origin. According to the clinical and pathology review, 41 samples were presumed to have a MPE while 24 patients BPE. The most common aetiology for MPE was lung adenocarcinoma (*n* = 36), in five cases the effusate was associated with a metastatic lung process of hepatic, ovarial, gastric, breast or renal primary carcinomas.

### Effusate cfDNA Yield and Fragment Sizes

Effusate cfDNA yields isolated from 5 ± 0.5 ml cell-free fraction ranged from 0.21 to 46.8ng/µl, the mean concentration was 17.23 ng/µl, indicating a broad variability in free DNA content. Correlation of effusate and plasma cfDNA concentrations from the same time points was possible in 13 cases. Here, the mean cfDNA concentration of the effusate was 23.2 ng/µl (range: 0.83–56), which was significantly higher than cfDNA isolated from the same amounts of PB plasma (4.8 ng/µl, range: 1.44–18.3) (*p* < 0.05). Pearson correlation analysis between the matched plasma and effusate cfDNA concentration did not provide relationship (r: 0.3, *p* > 0.05).

**FIGURE 1:**
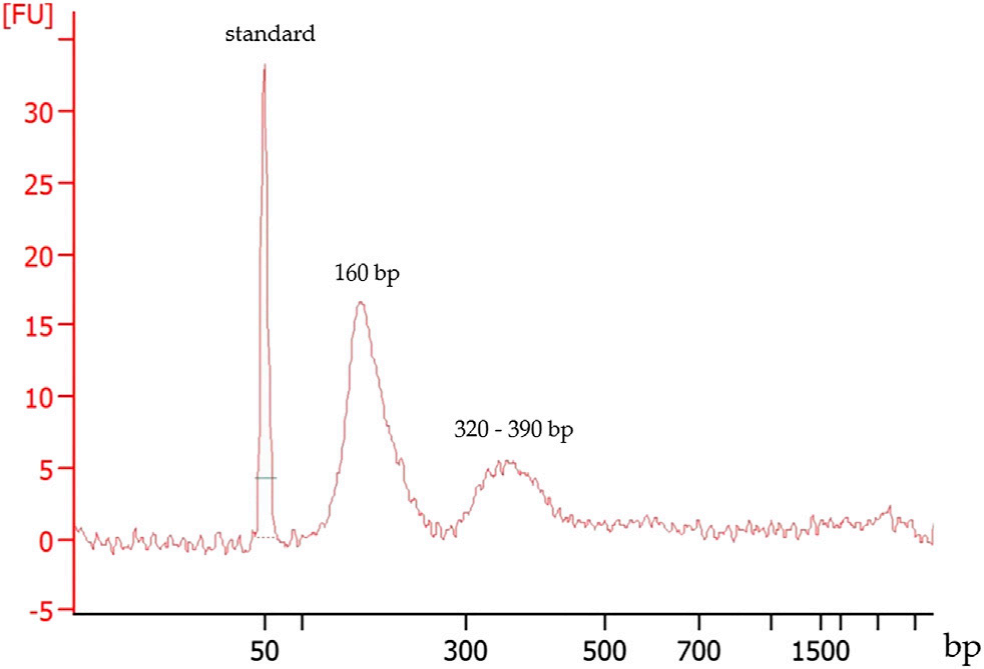
Typical fragment size distribution of a cfDNA extracted from a pleural effusion fluid. First peak: 50 bp marker, the second and the third peaks represent the cfDNA fragment sizes.

DNA size estimation demonstrated effusate cfDNA fragment size distribution around 160 bp and at a minor peak between 320 and 390 bp (Figure 1). This size distribution was in agreement with that observed for plasma cfDNA culminating around 165 bp.

**FIGURE 2:**
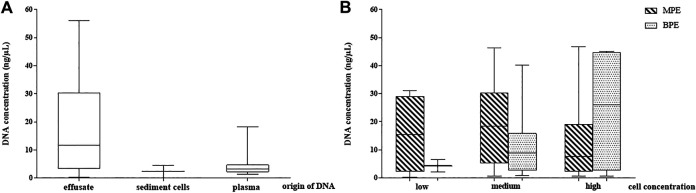
DNA concentration distribution in the study samples. **(A)** DNA yield in pleural effusion fluid (cell free DNA), sediment cells (genomic DNA) and periferial blood plasma cell free DNA. **(B)** Pleural effusion fluid DNA concentrations depending on cell concentration and their malignancy (observed microscopically). MPE: malignant pleural effusate, BPE: benign pleural effusate.

When comparing the yield of effusate cfDNA quantities with gDNA isolated from the sediment cell blocks separated from the same pleural fluid (performed in all samples, *n* = 65), significantly higher DNA amounts from the cell-free compartment were stated (17.23 vs. 2.4 ng/µl; r:0.81 *p* < 0.05). The relation of the DNA yield obtained from the effusate supernatant, the sediment cell block and the blood plasma from the same time is presented in Figure 2A.

Comparison of the MPE and BPE groups could not demonstrate significant differences in cfDNA concentration according to the gross origin of the fluid accumulation. The mean value for MPE was 19.25 ng/µl (range 0.21–46.8) and for BPE was 14.2 ng/µl (range 0.7–45.1).

To reflect the role of cellularity on the effusate cfDNA yield, three groups were created according to the cell mass represented by sediment cell-block microscopy (cell rich, medium, cell-poor). Mean cfDNA concentrations were 19.7 ng/µl, 18 ng/µl and 14.1 ng/µl, respectively (difference statistically not significant). No correlation between MPE pleural fluid cell content and the supernatant cfDNA concentrations could be stated, but the correlation analysis proved moderate significant relationship between low and high BPE samples (r:0.65, *p* < 0.05). Effusion fluid DNA concentrations depending on cell content and origin (MPE or BPE) are presented in Figure 2B.

### Comparative Mutational Profiling of Matched Sample Types in Lung Adenocarcinoma

As next we evaluated our targeted *EGFR*, *KRAS* and *BRAF* test results from cfDNA obtained from pleural effusion and blood and from gDNA isolated from sediment cell blocks and lung biopsies. The molecular genetic findings are presented in Table 2.

**TABLE 2:** Comparison of histological and molecular findings obtained from matched tumor and pleural effusion sample types from lung adenocarcinoma patients (# histological/cytological diagnosis established at an other institution, * patient refused bronchoscopy).

Patient	Sediment cell block cytology	Affected gene	Mutational status	Results of molecular testing			
				Tumor biopsy/cytology gDNA	Plasma cfDNA	Sediment cell block gDNA	Pleural effusion cfDNA
1	Adenocarcinoma	*EGFR*	c.2235_49del15, *p*.E746_A750del5	Not available #	Negative	Negative	Positive
2	Adenocarcinoma	*EGFR*	c.2573T > G, *p*.L858R	Not available #	Positive	Negative	Positive
3	Adenocarcinoma	*EGFR*	c.2240_2257del18, *p*.L747_P753delinsS	Not available #	Negative	Negative	Positive
4	Negative for malignancy	*KRAS*	c.34G > T, *p*.G12C	Positive	Positive	Negative	Positive
5	Adenocarcinoma	*KRAS*	c.34G > T, *p*.G12C	Not available #	Negative	Negative	Positive
6	Negative for malignancy	*KRAS*	c.34G > T, *p*.G12C	Positive	Negative	Negative	Positive
7	Negative for malignancy	*KRAS*	c.34G > T, *p*.G12C	Not available #	Positive	Negative	Negative
8	Adenocarcinoma	*KRAS*	c.34G > C, *p*.G12 R	Positive	Negative	Negative	Positive
9	Adenocarcinoma	*KRAS*	c.35G > T, *p*.G12 V	Positive	Negative	Negative	Positive
10	Negative for malignancy	*KRAS*	c.35G > C, *p*.G12 A	Positive	Negative	Negative	Positive
11	Adenocarcinoma	*KRAS*	c.179G > T, *p*.G60 V	Not available #	Negative	Negative	Positive
12	Adenocarcinoma	*KRAS*	c.183A > C, Q61H	Positive	Negative	Negative	Positive
13	Negative for malignancy	*KRAS*	c.34G > T, *p*.G12C	Not available *	Positive	Negative	Positive
		*BRAF*	c.1799T > A, *p*.V600 E		Positive	Negative	Positive

Using the reverse-hybridization technique pathogenic gene variants were only detected in MPE samples associated with primary lung carcinoma (13/36 cases, 36.1%), the other five lung metastasis related MPE and all 24 BPE cases remained negative for any of the gene mutations tested.

Three activating *EGFR* gene mutations were detected in the malignant pleural effusion samples (c.2235_49del15, p.E746_A750del5; c.2573T>G, p.L858R and c.2240_2257del18, p.L747_P753delinsS; 3/36, 8.3%) (Table 2, cases 1–3). Only one of them (c.2573T>G, p.L858R, case 2) was represented in the plasma cfDNA and was also confirmed in genomic DNA isolated from cytological smears obtained by bronchoscopy. The sediment cells were negative for *EGFR* mutation. Lung tissue biopsies were performed in other institutions in these cases and the samples were not provided for testing.

Six patogenic *KRAS* variants were found in an other set of the MPE samples (four c.34G>T, p.G12C; one c.34G>C, p.G12R, one c.35G>T, p.G12V; one c.35G>C, p.G12A; one c.179G>T, p.G60V and one c.183A>C, Q61H; 9/36, 25%). Out of the *KRAS* mutant cases (cases 4–13 in Table 2) only two matched plasma cfDNAs were positive for the same *KRAS* variants (cases 4 and 13). On the contrary, there was only one plasma liquid biopsy sample with a *KRAS* variant where the matching pleural effusion sample (both cfDNA and sediment) remained wild-type (case 7). No *KRAS* mutation could be identified in any of the sediment cell blocks. The corresponding *KRAS* variants were directly validated from all lung cancer tissues where a lung biopsy sample was available (cases 4, 6, 8, 9, 10 and 12).

In one of the included cases (case 13), an additional *BRAF* c.1799T>A, p.V600E mutation could detected beside the *KRAS* c.34G>T, p.G12C pathogenic variant (1/36, 2.8%) from the effusion cfDNA. Both variants appeared in the blood plasma cfDNA sample but not in the DNA isolated from the sediment cell block. Lung tumor biopsy sample was not provided as in this case as the patient rejected thoracoscopy.

**FIGURE 3:**
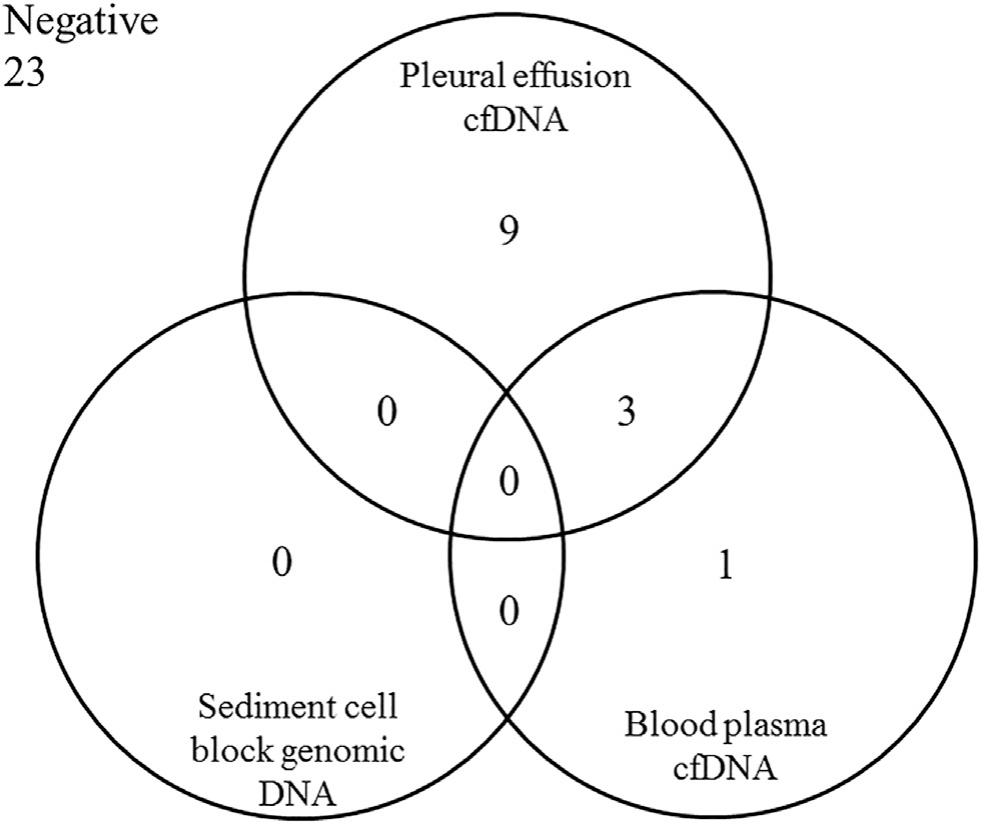
Distribution of the positive molecular genetic findings (pathogenic variant in *EGFR*, *KRAS* or/and *BRAF* genes) in the different sample types originated from the 36 patients with malignant pleural effusion fluid associated with lung adenocarcinoma.

The relation of the molecular genetic findings in the diverse sample types and fractions is presented in Figure 3.

## Discussion

Liquid biopsy, predominantly blood-based, is gradually integrating into molecular oncology practice, opening new perspectives when tumor sampling is complicated or when longitudinal sampling for treatment monitoring is required. Detection of mutations in cfDNA derived from plasma is an expanding and highly promising field, still with lot of challenges due to limited amounts and variability of cfDNA released in the circulation [14]. Alternative sources of cfDNA have also been considered, including body cavity fluids being now actively explored [15, 16]. Pleural effusions in malignancy can be considered as representants of tissue fluid anatomically and functionally related to tumor parenchyma. In theory, cancer related biomolecules – such as free DNA fragments - are continously released to the microenvironment in line with the progression. Evidence is growing that cfDNA isolated from the pleural fluid optimally reflects key biological processes making direct tumor sampling by complicated bronchoscopic/thoracoscopic interventions unnecessary.

Pleural fluid cytology had an important diagnostic value for longer times. Pleural effusate cytology for lung carcinoma cells had an average sensitivity of around 60% (from 40 to 87%) [17]. MPE is a frequent complication of lung carcinoma, which affects 40% of patients during the progression of the disease [18]. Minor fluid collections may indicate diagnostically challenging early or relapsed carcinoma and are subjects of careful sediment cytology (smears, cytospin preps). Alternatively, spinned cell pellets are fixed and embedded to obtain cell-blocks for specific identification of cancer cells by series of immunohistochemistry stainings. Pleural cytology/cell-block samples thus have been also considered for the molecular pathology practice, with known limitations [19–22].

While the cell-free supernatant of the pleural centrifugate was simply discarded earlier, its utility as an optional resource of DNA is recently under intensive studies. Our data revealed that the MPE supernatant is rich in cfDNA. Quantities of the isolated DNA were appropriate for molecular studies, and were actually significantly higher than obtained from the same volumes of PB plasma. Further, effusion supernatants provided more cfDNA for analysis than gDNA could be extracted from the matched cell block representing the cellular component of the effusion. In general, the quality of the MPE derived cfDNA enabled all kinds of genetic testing, comparable with the cfDNA isolated from the plasma.

Cellular degradation (e.g. due to ischemic necrosis) is a frequent feature of aggressive tumors releasing randomly and incompletly digested genomic DNA fragments of variable length [23], which basically differs from apoptosis derived nuclear DNA consisted of small nucleosomal fragments (<200 bp) [24]. In addition, malignancies may progressively excrete DNA fragments into the microenvironment in both free and exosomal fractions [25]. As a consequence, a spectrum of fragmented tumor-derived cfDNA accumulates in the tissue microenvironment and is carried away by the blood or the lymphatic circulation. The local redirection of the lymphatic flow in the proximity of the tumor may play a major role in the generation of pleural fluid and support continous release of cfDNA to the pleural cavity.

In the present study, quality and quantity issues of MPE cfDNA obtained from lung adenocarcinoma patients were addressed. Specific comparison of *EGFR*, *KRAS* and *BRAF* findings were compared following the analysis of tumor tissue, PB cfDNA, MPE cell pellets (cell-blocks) and cell-free MPE samples from the same probands. Priority was given to a simple and high-sensitivity reverse-hybridization assay, which was extended with Sanger sequencing and multi-gene NGS to validate the results. The positive rate for mutation detected in MPE cfDNA was satisfactory with 12 out of 13 mutant tumor patients tested (92.3%). This was consistent with other extended studies reporting on high sensitivity of sequencing in effusion cfDNA samples [26, 27]. Despite of the absence of novel, secondary variants within the current series (including the resistance mutation *EGFR* T790 M certainly covered by the reverse hybridization assay) we find this approach valid for follow up mutation testing.

To our surprise the sediment cell-blocks performed with disappointing efficacy in this comparison, that requires explanation. Three major causes were identified: 1) some of the pellet cell-blocks were completely negative for carcinoma cells after detailed cytological and immunohistochemistry evaluation, therefore, gene variants in the gDNA isolate could not be expected; 2) other cell-blocks with cytological positivity for malignancy presented with generally poor cellularity and DNA yields remained inappropriate for extended genetic testing; 3) pellet gDNA quantities enabled a single analysis in the rest of the cases which was done using the reverse-hybridization strip platform. Following the evaluation of all analytical factors no interference between the cell-block sample processing and the reverse-hybridization method could be identified. In contrast, inefficiency of cell-block derived gDNA testing basically relied in the low cell counts and in the inferior gDNA quantity provided for analysis. Further to the present data, another study also reported the superior performance of effusate supernatant cfDNA over the sedimentary malignant cells and PB cfDNA for variant *EGFR* detection, with significant underrepresentation of mutations in plasma cfDNA as well [28]. The results were explanined by the close anatomical proximity and by the special effect of tumor lymphatic drainage.

In conclusion, the cell-free fraction of MPEs is especially efficient for genetic testing compared to traditional cytological smears or cell block samples in cases with lung cancer. These results suggest that additional information may be generated from cfDNA of the saved pleural supernatant even when the routine protocol favours gDNA testing from cellular samples.

## Data Availability

The raw data supporting the conclusions of this article will be made available by the authors, without undue reservation.
